# Safer drug use in primary care - a pilot intervention study to identify improvement needs and make agreements for change in five Swedish primary care units

**DOI:** 10.1186/s12875-016-0542-8

**Published:** 2016-10-04

**Authors:** Sara Modig, Cecilia Lenander, Nina Viberg, Patrik Midlöv

**Affiliations:** 1 Department of Clinical Sciences in Malmö/Family Medicine, Lund University, Jan Waldenströms gata 35, SE-205 02 Malmö, Sweden; 2 Department of Medicines Management and Informatics in Skåne County, Malmö, Sweden; 3 Department for Public Health Sciences, Karolinska Institutet, Stockholm, Sweden

**Keywords:** Primary care, Medication safety, Peer-Review, Self-assessment, Elderly, Medication reconciliation

## Abstract

**Background:**

There is an urgent need to improve patient safety in the area of medication treatment among the elderly. The aim of this study was to explore which improvement needs and strengths, relating to medication safety, arise from a multi-professional intervention in primary care and further to describe and follow up on the agreements for change that were established within the intervention.

**Methods:**

The SÄKLÄK project was a multi-professional intervention in primary care consisting of self-assessment, peer-review, feedback and written agreements for change. Data were obtained from five primary care units randomised to the intervention group. Reviewer feedback reports and agreements for change were analysed using content analysis.

**Results:**

Strengths that were identified included a committed leadership, work methods to enhance medication safety and access to consultants. *Methods for securing an accurate medication list*, *knowledge and methods of working of the prescriber* and *patient’s ability to contribute to medication safety* were areas that gave rise to three predesigned categories for improvement needs on a local level. Another category became apparent during the analysis; namely learning from mistakes and from results. In all categories, apparent shortcomings were identified. These included inaccurate medication lists, lack of medication reconciliation, lack of time for follow-up of elderly patients, need for further education in geriatrics and pharmacotherapy and lack of information on indication and maximum dosage. An increased number of medication reviews were among the most common agreements for change seen.

**Conclusions:**

This study identified substantial shortcomings, like poorly updated medication lists, which affected medication safety in the participating Swedish primary care units. Similar shortcomings are most likely present in other primary care units in the country. Working together multi-professionally, including performing medication reviews, could be one way of improving medication safety. On the other hand, the individual physician must possess enough pharmaceutical knowledge and the working conditions must allow time for follow-up of prescriptions. Strengths of the primary care unit, such as successful methods of working, must be taken advantage of. The culture in primary care may affect the ability to successfully implement routines that improve patient safety and reduce risk of medication errors.

## Background

In Sweden, one in five people was aged 65 years or older in 2013 [1]. Elderly patients with multiple diseases often have several prescribers. With many different systems for documentation the risk of medication errors is apparent, especially when these elderly patients are transferred from hospital care to primary care for example [2, 3]. General practitioners (GPs) are central in this work since they often have overall responsibility for these patients. If GPs lack information about current drug use, they cannot take it into account when prescribing and the risk of adverse drug reactions increases. It is also essential to have routines that enable GPs to keep up to date in a number of therapy areas and in the knowledge of pharmacodynamics and kinetics in the elderly. The patient’s participation and compliance to prescription is of importance as well. Lack of compliance may result in increased morbidity and hence more health care consumption [4, 5].

A drug-related problem (DRP) is defined as an undesirable patient experience that involves drug therapy and that actually or potentially interferes with the desired patient outcome [6]. DRPs are one of the most common reasons for patient injury in health care. Elderly patients are especially vulnerable due to organ changes [7]. Besides the human suffering, DRPs are costly. As much as 35 % of unplanned hospitalisations among the elderly are potentially caused by DRPs [8]. The vast majority of these are avoidable [9, 10].

The Swedish National Board of Health and Welfare has come to the conclusion that continuity in the contact between patient and physician is needed in order to improve quality and safety in the area of drug safety among the elderly. Furthermore, physicians must have enough knowledge, be accessible and have enough time [11].

### Aim

The aim of this study was to explore which improvement needs and strengths, relating to medication safety, arise from a multi-professional intervention in primary care. Major intervention measures were self-assessment and peer-review. Furthermore, the aim was to describe and follow-up on the written agreements for change that were settled within the intervention.

## Methods

### Setting

The SÄKLÄK intervention project was initiated by the Swedish Association of Local Authorities and Regions (SALAR) and the Swedish National Patient Insurance Company LÖF. The steering committee of the project consisted of one delegate each from six professional organisations (The Swedish College of General Practice, The Swedish Pharmaceutical Society, Geriatric Medicine in Sweden, the Swedish association of Local Authority Senior Medicine Advisors, Sweden’s National Organisation of District Nurses and The Swedish Society of Clinical Pharmacology and Therapeutics). The SÄKLÄK project was a pilot study to determine whether a method developed for hospital care [12, 13] could be used in primary care to enhance medication safety in elderly patients. Invitations to participate were emailed to all primary care units in Sweden in 2013. Participation was open to all. A total of 20 units applied and they were stratified according to urban or rural location. A random sample of 10 units was drawn using Excel. Five units were randomised to the intervention group and five to the control group, keeping the distribution between urban and rural units. The units took part voluntarily and without financial compensation.

### The intervention model

The SÄKLÄK project, which was carried out in 2013/14, was a multi-professional intervention in primary care consisting of self-assessment, peer-review, feedback and written agreements for change. The project was addressed to the head of the primary care unit, with whom a written agreement was made to accomplish every step in the process. No specific improvement method was supplied by the project.

In short, the intervention started with a self-assessment questionnaire with questions on how patient safety is secured during prescribing of medication, medication use and follow-up. It dealt with safety at the primary care unit as well as in co-operation with pharmacies, hospitals and municipally provided home care. The head of the primary care unit was responsible for the self-assessment, which was carried out in co-operation with co-workers at the unit as well as cooperating caregivers. See below for the content of the questionnaire. A select group of doctors, nurses and pharmacists served as reviewers and visited the primary care units in the second step of the intervention. The reviewers fulfilled their obligations supported by written instructions, documents and continuous contact with the project management. Thereafter the reviewers produced a written feedback report for the primary care unit. The reviewers and the management at the primary care unit then agreed on an action plan for improvements. These agreements, five to 10 per unit, were chosen among the improvement suggestions in order to be possible to fulfil within the 6-month follow-up time. The procedures of the intervention model are described in Table 1.

**Table 1:** Description of the different parts of the intervention model (SÄKLÄK)

1. Introductory meeting	Representatives from the steering committee^a^ visited the primary care units, gave a structured introduction to the intervention model for unit managers and staff representatives, including nurses working in home care and pharmacists. The involvement of all professional categories was presented as a prerequisite for the self-assessment process.
2. Structured self-assessment	The self-assessment questionnaire contained 12 questions covering areas of importance for safe use of medications in primary care, with focus on elderly patients with multiple diseases. The head of the primary care unit was responsible for the self-assessment, which was carried out together with all professional categories as well as cooperating caregivers.
3. Peer review	A group of physicians, nurses and pharmacists selected by the professional organisations^b^ served as reviewers. For each primary care unit, a multi-professional team consisting of five to six reviewers was formed, which discussed the answered self-assessments. The primary care units were visited by a peer-review team. A document based on the questions used in the self-assessment procedure served as support for the peer review where new or updated information arising during the visit was noted.
4. Written feedback and agreement for change	The peer-review team presented a written feedback report regarding their view on strengths and weaknesses, priority areas for improvement and proposed measures to be taken. Eventually, a written contract consisting of a detailed action plan was jointly produced by the primary care unit and the peer-review team.
5. Follow-up seminar	A seminar for the steering committee, the reviewers and all managers at the intervention primary care units.
6. Follow up on accomplishment of agreements	The agreements for change were to be followed up on 6 months after they were signed.

^a^The steering committee comprised representatives from the Swedish Association of Local Authorities and Regions (SALAR) and The Swedish National Patient Insurance Company LÖF and one delegate each from the six professional organisations listed below

^b^The Swedish College of General Practice, The Swedish Pharmaceutical Society, Geriatric Medicine in Sweden, Riksföreningen för Medicinskt Ansvariga Sjuksköterskor (a Swedish association of authorized nurses), Sweden’s National Organisation of District Nurses and The Swedish Society of Clinical Pharmacology and Therapeutics

### The self-assessment questionnaire

The self-assessment questionnaire was developed by a multi-professional expert group, which was elected by the professional organisations. It was web-based and constructed in order to promote multi-professional co-operation in the assessment process. The questionnaire consisted of 12 questions that focused on how the primary care unit secured the following:

1. The prescriber has complete and correct information about the patient’s medication use.
2. The treatment with medications is well motivated in the light of the patient’s diseases and individual conditions.
3. The medications do not interact in a harmful way.
4. The environmental effects of the medications are reduced to a minimum.
5. The patient is able to handle his/her medications.
6. Medication treatment is followed up and evaluated.
7. The co-operation within hospital and specialised care is satisfactory.
8. The co-operation with the municipally provided home care is satisfactory.
9. The co-operation with the local pharmacies is satisfactory.
10. The organisation learns from negative incidents.
11. Medication reviews are carried out.
12. Other local methods or routines which contribute to safe drug use.

For each of the questions, five further follow-up questions were asked:

- a. What methods/routines/guidelines do you have?
- b. How do you provide conditions to ensure compliance?
- c. How do you measure compliance?
- d. How do you give feedback on the results to the staff?
- e. What ideas do you have for improvement?

### Analysis

Data from the intervention group, i.e., reviewer feedback reports and written agreements for change, generated a large amount of information and were analysed using three different approaches to content analysis, as described by Hsieh and Shannon [14] among others. Content analysis initially became renowned as a quantitative method in the field of media research, where it was used to describe text data with the help of statistics [15]. Over time it has evolved into different qualitative methods that have come into widespread use in health research [14].

### Deductive and inductive content analysis

Units of analysis were reviewer feedback reports. The first author, who is a primary care physician with solid experience in primary care and medication safety, defined a number of categories that describe crucial areas of improvement to attain medical safety. These pre-defined categories were based on the self-assessment questionnaire that was developed by an expert team to contain the most important topics for medication safety. During the deductive content analysis, the material was read through several times by the first author in order to get acquainted with the content. Text units relating to the same topic were identified and sorted under the respective pre-defined category for improvement needs. Studying the units of text under each category yielded different sub-categories that described specific methods used in primary care, and in co-operation with other care givers, to attain medication safety. Some of the improvement needs were specifically linked to the regional or national level and have therefore been presented under a separate heading. Furthermore, during the analysis a number of strengths were also identified that gave rise to five categories. This part of the analysis can hence be described as an inductive content analysis [14].

### Summative content analysis

The written agreements for change could all be sorted under the same headings as the predefined categories of improvement needs, except for a few of general character. The number of times an agreement related to a certain topic was settled was calculated and presented in a table. Whether the agreement was fulfilled or not within follow up-time was also noted.

## Results

**Fig. 1:**
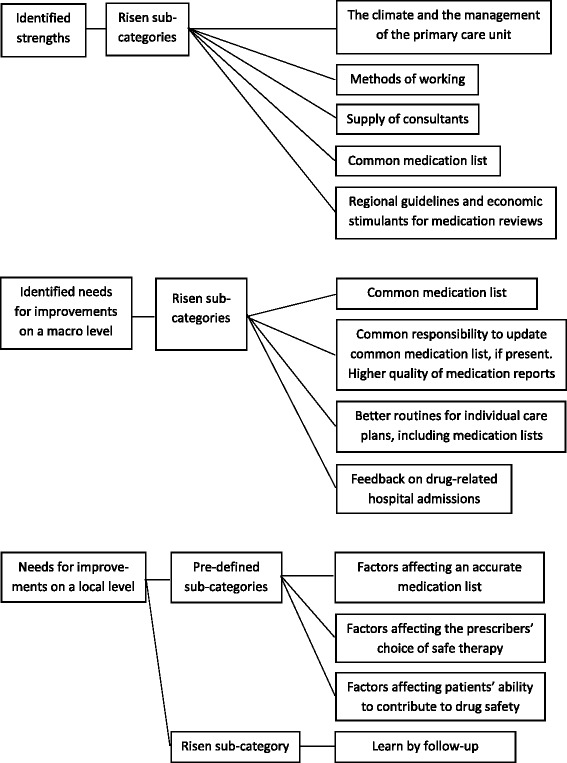
Illustration of the pre-defined categories for improvement need, the risen sub-categories and the identified strength categories

The intervention with self-assessment and peer-review identified several local and regional strengths which contributed to safer drug use, a large number of needs for improvement on both a local and regional/national level, and yielded different kinds of agreements for change. The predefined categories for improvement need, the risen sub-categories and the identified strength categories are presented in Fig. 1.

### Strengths

The analysis yielded five categories of strengths that were identified by the reviewers and primary care unit self-assessment and present in various extensions between the units:

#### 1. The culture and the management of the primary care unit

An open climate for discussion and a committed leadership with a positive attitude towards development was identified as a strength in the work for improvement.

#### 2. Methods of working

Some of the units had created routines and arranged ways of working that led to improved medication safety, for example regular meetings giving possibilities for physicians to receive guidance from colleagues, having only experienced GP responsibility for nursing homes, enough time for physician appointments and specially appointed nurses for elderly patients with multiple illnesses. Regular assessment of cognitive and physical function of the patients was used to guarantee patients’ ability to manage their drug treatment. Routines for evaluation of results included regular random checks that medication changes were satisfactorily documented in the medical records and feedback on prescription trends. Always adding the medication list to referrals implied a necessity and demand on a continuously updated and accurate list.

#### 3. Supply of consultants

Some units received consultant help from other specialities or from the regional drug and therapeutic committee such as geriatricians, psychiatrists or pharmacists.

#### 4. Common medication list

A common medication list, used both by specialised care and primary care, was identified as a strength since it allowed all prescribers to be aware of every prescription and take it into account.

#### 5. Regional guidelines and economic stimulants for medication reviews

Guidelines and economic incentives for medication reviews were present in some regions and were identified as a strength and a method to increase medication safety.

### Needs for improvement on a macro level

The needs for improvement, which have to be handled on a regional or national level, gave rise to four sub-categories:

#### 1. Common medication list

All regions did not have a common medication list shared by primary care, hospital care and even municipal care, or did not even have the possibility to read each other’s medical records. This was identified as an obvious risk for potential duplicated prescriptions, interactions etc.

#### 2. Updated medication lists - everybody’s responsibility

Even if a common list was present the maintenance of the list was often unsatisfactory. For example, dosage changes could be documented in the medical record but not carried out in the medication list. Neither was the medication report [3] always correct and municipal nurses used a lot of time to obtain an accurate prescription list when the patients returned from hospital care.

#### 3. Better routines for individual care plans, including medication lists

The routines for setting up individual care plans, especially after hospital care, did often not include taking the medication list into account, which was identified as an obvious lack of an important part of the care.

#### 4. Feedback on drug-related hospital admissions

There were no routines found for giving feedback to primary care on drug-related hospital admissions. Reviewers noted that such feedback would be a knowledge resource and could influence handling in primary care.

### Needs for improvement on a local level

As safer drug use is dependent on methods for securing an accurate medication list, knowledge and methods of working of the prescriber, and patient’s compliance; these were the three predesigned categories for different kinds of improvement needs on a local level. A fourth area became apparent during the analysis, namely learning from mistakes and from results.

#### Pre-defined category 1: Factors affecting an accurate medication list

Improvement methods which contributed to a more accurate medication list gave rise to three sub-categories:

#### 1a. Update of the medication list

Routines for constantly updating the medication list were not always present. Reviewers noted that temporary physicians and locum tenens must also be informed to ensure that such routines are followed.

#### 1b. Medication reconciliation

Many suggestions for introducing different routines for medication reconciliation emerged. The reviewers noted that the physician should always ensure that the medication list in the record corresponds to what the patient actually uses during the appointment. A clinical pharmacist, or a nurse, might meet or talk to the patient before the doctor appointment in order to secure the list. Routines must also exist for always asking for OTC (over the counter) drugs or herbal drugs.

#### 1c. Medication reports

Introduction of medication reports have been directed by the National Board of Health and Welfare, but were still not implemented everywhere. The need for such a report was obvious since the IT systems do not automatically report medication changes that have been made at other care units. The reviewers also noted that reports, in the form of a copy of the medical record, should always be sent to the district nurse when municipal care patients have visited the primary care unit.

#### Pre-defined category 2: Factors affecting the prescribers’ choice of safe therapy

Improvement methods which contribute to a safer choice of therapy by the prescriber gave rise to five sub-categories:

#### 2a. Medication reviews

Work with medication reviews was established at every unit, but both quality and quantity of the medication reviews were considered to have to increase. Not all patients covered by the criteria were offered medication reviews. Needs of a changing culture among parts of the GP group in their attitude towards medication reviews were also seen.

#### 2b. Follow-up of prescriptions

Every patient, who was prescribed regular medication, was not offered regular appointments with his/her GP. This was identified as a decreased possibility for follow-up of prescriptions. The name and dosage of the medication was often documented in the records, but it was not always accompanied by the indication for treatment and/or a follow-up plan. No systematic follow-up of adverse drug reactions were found.

#### 2c. Education and discussion/consensus between colleagues

Further education in geriatrics and pharmacotherapy should be offered to GPs and other staff on a more regular and extensive basis. Bringing about a consensus among GPs about drug prescriptions and inappropriate medications for the elderly was considered important by the reviewers.

#### 2d. Need for prioritisation of the frailest patients

The time GPs spent doing consultations at nursing homes was sometimes considered not to be enough. The reviewers noted that working for better possibilities for the regular GPs to accomplish acute visits in the patients’ homes, and thereby minimise external physician actions, would increase continuity and thereby most likely the medication safety by better follow-up.

#### 2e. Need for routines for noting deviation from recommended prescription

The reviewers noted a lack of a routine for noting in the medical record when drugs are prescribed, despite an interaction with existing prescriptions. They also noted that this should be routinely made even when potentially inappropriate medications are prescribed.

#### Pre-defined category 3: Factors affecting patients’ ability to contribute to drug safety

Improvement needs which affect the patients’ contribution to safety gave rise to three sub-categories:

#### 3a. Accurate medication list to rely on

The patient should always be supplied with an updated medication list in connection with an annual appointment at the primary care unit. Routines for medication reconciliation prior to a visit, i.e., by a nurse or by asking the patient to bring a list from the National Pharmaceutical Register should always exist. Routines should also be developed for having all outdated prescriptions cancelled and all drugs from finished treatments returned to pharmacies.

#### 3b. Information on indication and maximum dosage

The indication for treatment should always be known for the patient as well as the follow-up plan. However, this was not always the case. A routine for informing patients about the reasons for the prescriptions might help. To arrange feedback from the local pharmacy about prescriptions without indication or maximum dosage for “as needed” would increase awareness among GPs.

#### 3c. Direct patient support to enhance compliance

The reviewers noted that an increased involvement of the district nurse to ensure that patients can open drug packaging and use prescribed medication would increase compliance.

#### Pre-defined category 4: Learn by follow-up

The reviewers noted that both follow-up of mistakes in the medication area and evaluating outcomes, i.e., compliance to therapy recommendations, should be done more extensively since it contributes to awareness and knowledge. As it stands currently the reporter of mistakes does not get any feedback on less serious events.

### Agreements for change

A total of 38 agreements for change were concluded between the heads of the primary care units and the peer-review teams. These were divided into the same categories as the improvement needs; see Table 2. Among the 38 agreements, 10 dealt with the need for increased number of medication reviews.

**Table 2:** Type and number of agreements between the peer-review teams and the primary care units

Main area for agreement	Number of agreements (*N* = 38)	Agreements not fulfilled within follow-up time (*n* = 12)^a^
Updating the medication list (1a)	2	
Medication reconciliation (1b)	4	2
Medication reports (1c)	1	
Medication reviews (2a)	10	2
Follow-up of prescriptions (2b)	2	
Education and discussion/consensus between colleagues (2c)	4	2
Need for prioritisation of the frailest patients (2d)	1	1
Accurate medication list to rely on (3a)	1	
Information on indication and maximum dosage (3b)	3	1
Follow-up of mistakes (4a)	2	1
Follow-up of results (4b)	1	2
Agreements which have to be taken care of on a regional or national level	3	
General character	4	1

^a^included in the 38 agreements

The primary care units had not managed to meet 12 of them when the agreements for change were followed up 6 months after they were signed. No obvious trends among the omissions were observed. Several dealt with local routines which still were not introduced. One agreement needed regional measures and two were co-operative actions.

## Discussion

This study confirmed that medication safety in the investigated Swedish primary care units has substantial shortcomings. To achieve safe drug use it is required that an accurate medication list is always provided. Every involved prescriber must always update the list and be aware which other medications one has to take into account when making new prescriptions. Furthermore, it is essential that the prescriber’s knowledge is up-to-date regarding safe drug treatment in the elderly and that his/her ways of working enhance medication safety. Last but not least, the patient must be given a fair chance to achieve compliance and act in a safe and informed way. Frail elderly people are more likely to experience significant errors. Targeting the more susceptible population groups and the most dangerous aspects of the system may be an effective approach to error management and prevention [16].

A large number of improvement needs are identified in the project. These are often of basic art, implying that extensive measures are needed. The gap between desirable and existing conditions regarding drug safety was found to be larger than has been identified in the other projects using the same method [12, 13]. These projects have been performed in hospital clinics (obstetric, orthopaedic and abdominal surgery) where it might be easier to implement routines than in primary care with many small and isolated units. An important finding in this study is that the prevailing culture at the primary care unit can affect medication safety, both in positive and negative directions. The culture within primary care may sometimes be characterised by “working according to one’s own standards” and routines therefore might have less impact [17, 18]. Many national and regional guidelines already exist but this project reveals that the primary care units do not always use them. The management is often not leading the medical work in primary care, as it is in hospital clinics, even though the primary care unit management must create the conditions for work to function at the prescriber/patient level. Moreover, a committed leadership that is positive for development was found to be a strength in the work for safer medicine management [19]. Among the agreements, which were not fulfilled within the follow-up time measures, that would be rather easy to carry out locally were also present. This raises the question whether other commitments and orders are prioritised instead, for example availability or financial incentives?

Shortcomings regarding medication reconciliation were obvious in this project. This was one of the most important risk factors for insufficient medication safety among the investigated units and most likely also for Swedish primary care in general. Updating the medication list in the medical record is often done deficiently, which has been noted previously [20, 21]. Medical records that do not accurately reflect the patient’s current medication list are open invitations to possible significant medical errors. However, a Swedish qualitative study among GPs found variation regarding understanding about who is responsible for the patient’s medication list and in the use of different strategies to manage this responsibility [22]. This may complicate quality improvement work. Furthermore, there are different interpretations of the meaning of medication reconciliation and varying perspectives about the purpose [23]. The reconciliation process should, however, be standardised and implemented in daily practice as a routine part of the work in primary care [24]. Medication reconciliation is, according to the Swedish National Board of Health and Welfare, a mapping of all prescribed and used medications and a control whether the documented medication list is accurate in order to provide both care giver and patient an updated overall picture. The reconciliation should include OTC drugs as well. Medical trainees are often responsible for medication reconciliation on admission, transfer and discharge of the most vulnerable patients. Therefore, education on this aspect is important [25].

Another need for education was highlighted by the reviewers; namely geriatric and pharmaceutical further education. Several agreements for change dealt with this necessity. It has been suggested that the prescribing stage is the most susceptible for errors and elderly patients are more likely to experience significant errors [16]. This implies a demand for sufficient education in pharmacotherapy, since prescribing is an important part of every GP’s daily work. It is evidence-based that education and information for physicians leads to decreased prescription of inappropriate medications for the elderly [26].

Medication reviews are an important part of the medication safety process and involve several components of the work, i.e., medication reconciliation, evaluation of prescriptions and often co-operation with municipal care. Medication reviews were often highlighted in the project as a quality improvement method. Of the 38 agreements, 10 dealt with the need for an increase in both number and quality of the medication reviews. This might be due to the fact that medication reviews are easy to follow-up in number and therefore fit the project design but hopefully the high share of agreements rest on the fact that medication reviews are evidence based and can, together with other interventions, lead to improved safety [27]. This also applies to primary care even if the literature in this field is sparser [28–30].

Although the multi-professional approach was emphasised as very positive by the participants [31], this method of medication safety improvement was not prominent among the agreements. One reason may be that this takes time but according to the participants it could be seen as an investment. Multi-professional co-operation also gives an opportunity to share knowledge [31]. However, talking about teamwork might be nice and perhaps even correct, but when it comes to measures in reality one thinks more in terms of specific actions. This is in spite of the fact that individual interventions have only demonstrated marginal improvements in medication safety when implemented on their own [16]. Perhaps it is too challenging and overwhelming to affect organisational changes? Where to start and who to initiate? One way of working in a multi-professional way when it comes to municipal care patients, however, is to perform medication reviews. A multidisciplinary approach, targeting several different areas including medication reviews, improves medication safety and has been shown to substantially lower readmissions [32].

A common medication list shared by primary care, hospital care and sometimes even municipal care was viewed both as a strength and also as a problem. However, the shared list was often found not to be kept up to date and contained faults, as was also seen in a Swedish thesis from 2010 [33]. Taking responsibility to review all the patient’s medications was perceived as important but described as still not being done. The physicians did not make needed changes to the list of medications due to different barriers which rested both on individual physicians and on the system. A continuously updated common list would of course be considered as a strength and contribute to a better survey of prescriptions and improved safety.

**Fig. 2:**
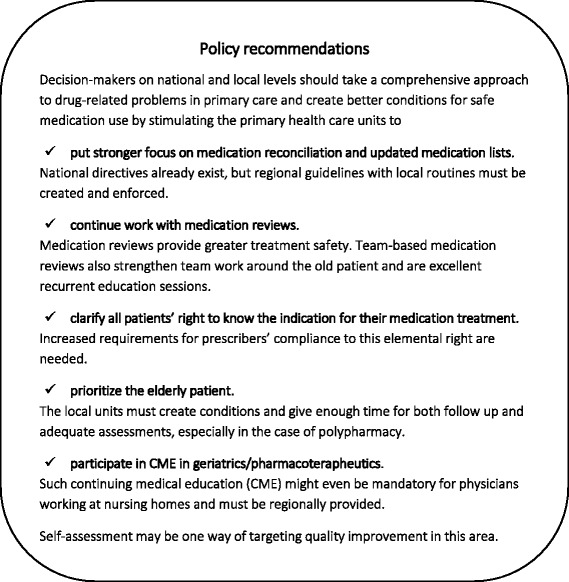
Policy recommendations for creating better conditions for safer drug use in Swedish primary care

Other strengths which were found in the project, where some of the primary care units had already come far in their efforts for medication safety, are important to take advantage of. Methods of working that prioritise frail elderly, that give enough time for guidance from colleagues and adequate further education, and that allow learning from feedback and results should not be kept within their own unit. The findings from the SÄKLÄK project are planned to be spread nationally and the method will be further developed and tried out. Different models for improving medication safety in the elderly population need to be compared in parallel. Policy recommendations for creating better conditions for safer drug use in Swedish primary care, based on the findings in this study, are presented in Fig. 2.

The SÄKLÄK project was a pilot study with few included units. We cannot deduce whether the findings from the selected units are representative of Swedish primary care or not, even though participation was voluntary and open to all primary care units in Sweden. This might entail that participating units had an extra interest in medication safety. In spite of that possible selection bias, we found substantial shortcomings. Furthermore, since the study was performed in a Swedish context we do not know if the findings can be applied to primary care in other countries.

The categorisation was discussed among the authors until consensus was reached. Two reviewers in the project (GPs) validated that the findings originated from the material and that the discussion is in line with the findings.

## Conclusions

This study identified substantial shortcomings, such as poorly updated medication lists, which affect medication safety in the participating Swedish primary care units. Although this is a pilot study conducted in a limited number of units, similar shortcomings are most likely present in other primary care units in the country. Working together multi-professionally, including performing medication reviews can be one way of targeting medication safety. On the other hand, the individual physician must possess enough pharmaceutical knowledge and the working conditions must allow time for follow-up of prescriptions. Strengths of the primary care unit such as successful methods of working must be taken advantage of. The culture in primary care may affect the ability to successfully implement new routines that improve patient safety and reduce risk of medication errors.

## Abbreviations

GP: General practitioner
DRP: Drug related problem
SÄKLÄK project: Safer drug use in primary care project
SALAR: Swedish Association of Local Authorities and Regions
OTC: Over the counter
